# Landscape of germline pathogenic variants in patients with dual primary breast and lung cancer

**DOI:** 10.1186/s40246-023-00510-7

**Published:** 2023-07-17

**Authors:** Ning-Yuan Lee, Melissa Hum, Sabna Zihara, Lanying Wang, Matthew K. Myint, Darren Wan-Teck Lim, Chee-Keong Toh, Anders Skanderup, Jens Samol, Min-Han Tan, Peter Ang, Soo-Chin Lee, Eng-Huat Tan, Gillianne G. Y. Lai, Daniel S. W. Tan, Yoon-Sim Yap, Ann S. G. Lee

**Affiliations:** 1 Division of Cellular and Molecular Research, National Cancer Centre Singapore, 30 Hospital Boulevard, Singapore, 168583 Singapore; 2 Division of Medical Oncology, National Cancer Centre Singapore, 30 Hospital Boulevard, Singapore, 168583 Singapore; 3 SingHealth Duke-NUS Oncology Academic Clinical Programme (ONCO ACP), Duke-NUS Medical School, 8 College Road, Singapore, 169857 Singapore; 4 Genome Institute of Singapore, 60 Biopolis St, Singapore, 138672 Singapore; 5 Medical Oncology Department, Tan Tock Seng Hospital, 11 Jalan Tan Tock Seng, Singapore, 308433 Singapore; 6 Johns Hopkins University, Baltimore, MD 21218 USA; 7 Lucence Diagnostics Pte Ltd, 211 Henderson Road, Singapore, 159552 Singapore; 8 Oncocare Cancer Centre, Gleneagles Medical Centre, 6 Napier Road, Singapore, 258499 Singapore; 9 Department of Hematology-Oncology, National University Cancer Institute, Singapore (NCIS), National University Health System, 5 Lower Kent Ridge Road, Singapore, 119074 Singapore; 10 Cancer Science Institute, Singapore (CSI), National University of Singapore, 14 Medical Dr, Singapore, 117599 Singapore; 11 Clinical Trials and Epidemiological Sciences, National Cancer Centre Singapore, 30 Hospital Boulevard, Singapore, 168583 Singapore; 12 Department of Physiology, Yong Loo Lin School of Medicine, National University of Singapore, 2 Medical Drive, Singapore, 117593 Singapore

**Keywords:** Multiple primary cancers, Breast cancer, Lung cancer, Whole-exome sequencing, Germline variants

## Abstract

**Background:**

Cancer predisposition is most often studied in the context of single cancers. However, inherited cancer predispositions can also give rise to multiple primary cancers. Yet, there is a paucity of studies on genetic predisposition in multiple primary cancers, especially those outside of well-defined cancer predisposition syndromes. This study aimed to identify germline variants associated with dual primary cancers of the breast and lung.

**Methods:**

Exome sequencing was performed on germline DNA from 55 Singapore patients (52 [95%] never-smokers) with dual primaries in the breast and lung, confirmed by histopathology. Using two large control cohorts: the local SG10K_Health (n = 9770) and gnomAD non-cancer East Asians (n = 9626); and two additional local case cohorts of early-onset or familial breast cancer (n = 290), and lung cancer (n = 209), variants were assessed for pathogenicity in accordance with ACMG/AMP guidelines. In particular, comparisons were made with known pathogenic or likely pathogenic variants in the ClinVar database, pathogenicity predictions were obtained from in silico prediction software, and case–control association analyses were performed.

**Results:**

Altogether, we identified 19 pathogenic or likely pathogenic variants from 16 genes, detected in 17 of 55 (31%) patients. Six of the 19 variants were identified using ClinVar, while 13 variants were classified pathogenic or likely pathogenic using ACMG/AMP guidelines. The 16 genes include well-known cancer predisposition genes such as *BRCA2, TP53,* and *RAD51D;* but also lesser known cancer genes *EXT2*, *WWOX*, *GATA2*, and *GPC3.* Most of these genes are involved in DNA damage repair, reaffirming the role of impaired DNA repair mechanisms in the development of multiple malignancies. These variants warrant further investigations in additional populations.

**Conclusions:**

We have identified both known and novel variants significantly enriched in patients with primary breast and lung malignancies, expanding the body of known cancer predisposition variants for both breast and lung cancer. These variants are mostly from genes involved in DNA repair, affirming the role of impaired DNA repair in the predisposition and development of multiple cancers.

**Supplementary Information:**

The online version contains supplementary material available at 10.1186/s40246-023-00510-7.

## Introduction

Multiple primary cancers (MPCs) refer to two or more malignancies arising independently in different organs of an individual that are not due to recurrence or metastasis. MPCs occur at a frequency of between 2 to 17% within 20 years of follow-up [1]. An estimated 1% (6,269/620,429) of female primary breast cancer patients develop primary lung cancer, based on data obtained from the Surveillance Epidemiology, and End Results (SEER) Program [2]. Genetic susceptibility, malignancy resulting from previous anticancer treatments, lifestyle factors such as smoking and alcohol consumption and environmental influences are some factors that increase the risk for MPCs [1]. For example, breast cancer patients who have undergone radiotherapy are at an increased risk of having a second malignancy of lung cancer [3, 4].

MPCs occur in various combinations, such as breast and ovarian cancer in hereditary breast and ovarian cancer [5] or colon and endometrial cancer in Lynch syndrome [6]. However, for other MPCs, such as for breast and lung cancer, there is a paucity of information on candidate cancer predisposition genes. To date, there has been a study that has identified *GSN* as a candidate gene significantly associated with the development of lung cancer in patients with a history of breast cancer, based on germline whole-exome sequencing (WES) of 28 cases [7].

In a landmark study on MPCs, whole genome sequencing (WGS) was performed on 460 individuals from 440 families to identify variants in 83 known cancer-predisposition genes. Tumor combinations included breast and colorectal cancer (51/883; 5.8%); breast and ovarian (34/883; 3.9%); breast and thyroid (23/883; 2.6%); and breast and lung cancer (12/883; 1.4%) [8]. Pathogenic variants were identified in moderate- and high-risk cancer predisposition genes in 15.2% of probands (67/440), but none of these patients had been diagnosed with primary breast and lung cancer [8]. More recently, a study has assessed genetic susceptibility to MPCs (n = 6429) across 36 organ sites, through WES of two multi-ancestry study populations [9]. A total of 22 variant-phenotype associations were identified, which included rare and common variants. Gene-based burden test showed that 9.52% and 6.78% of individuals with breast and lung cancer had predicted loss of function variants in *BRCA1* and *BRCA2*, respectively. Hence, there are few in-depth WGS or WES studies focusing on the combination of primary malignancies of the breast and lung. The rationale for this study was to elucidate the underlying genetic predisposition to account for the occurrence of these dual malignancies.

**Fig. 1:**
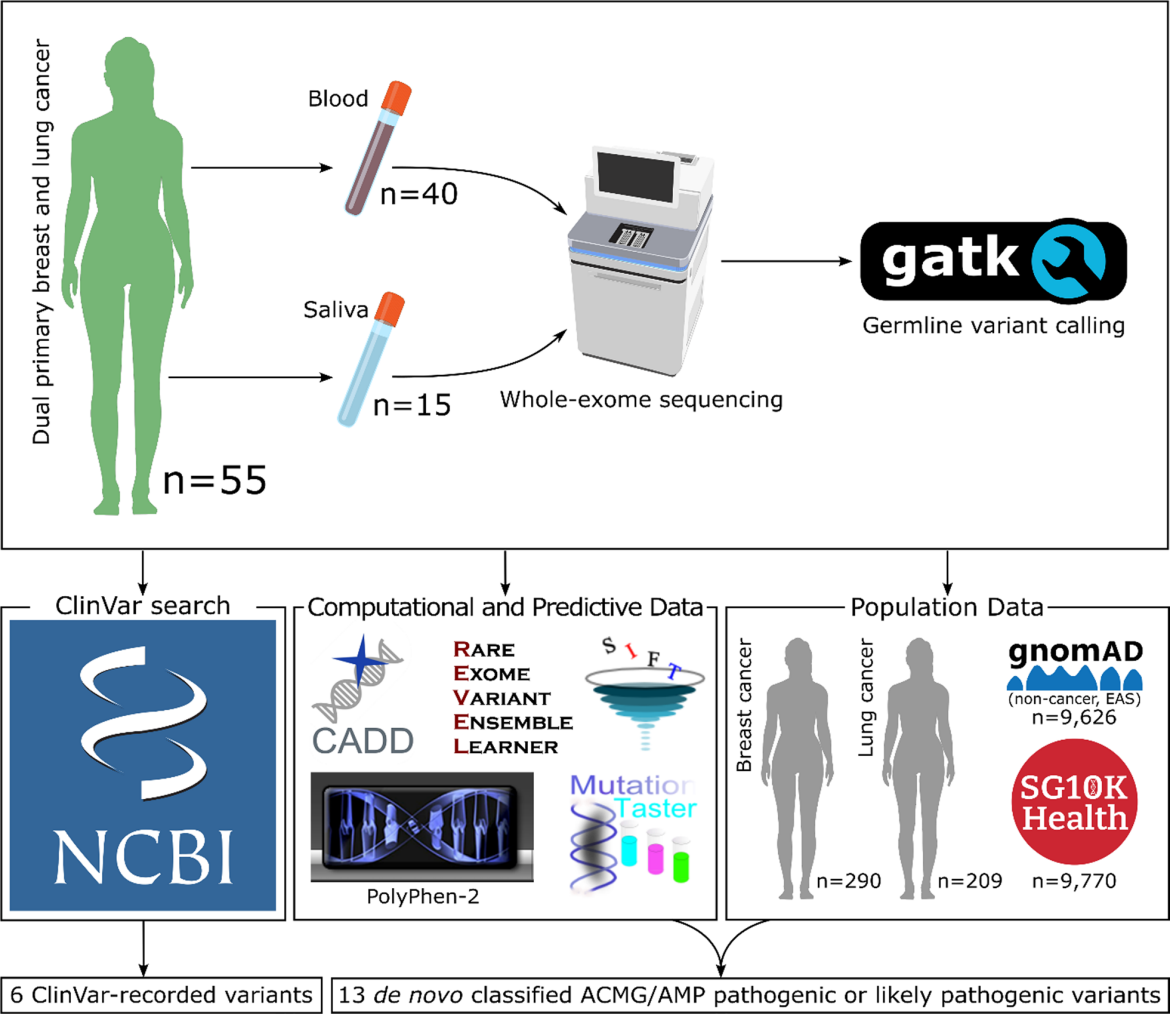
Overview of the study design

Given that lung cancer is one of the most common second primary in breast cancer patients [10], we have performed WES on germline DNA from a cohort of 55 patients with both primary breast and primary lung cancer to identify candidate predisposition variants. Variants were classified as pathogenic or likely pathogenic for cancer predisposition in accordance with ACMG/AMP guidelines, using annotations from the ClinVar database or by evaluating the guidelines. The overall study design is illustrated in Fig. 1. We have identified 19 pathogenic or likely pathogenic variants in 16 genes, detected in 17 of our 55 (31%) patients. Most of these 16 genes are well-known and involved in DNA damage repair, such as *BRCA2, TP53,* and *RAD51D.*

## Results

### Clinicopathological characteristics of our study cohort

Information on the demographics, age of onset, ethnicity, family history, and clinicopathological characteristics of the 55 patients with primary breast and lung cancers is provided in Table 1. A large proportion of patients in our study (94.5%) never smoked. Slightly more than half (54.5%) of the patients’ breast and lung cancers occurred at the same right or left side. Thirty-eight (69.1%) subjects developed breast cancer before lung cancer; radiotherapy to the breast or chest wall had been administered to 11 of them, with 6 of these patients having ipsilateral breast and lung cancers. Six (10.9%) patients had bilateral breast cancers and four (7.3%) patients had synchronous dual lung primaries, including one with bilateral breast cancer. Of the 45 lung adenocarcinoma never-smoker patients with known EGFR status, 33 (73.3%) had EGFR-mutated tumors (Table 1). We did not observe an association between hormone receptor status with *BRCA1*, *BRCA2* or *EGFR* mutation status in lung adenocarcinoma patients (Additional file 1: Table S1).

**Table 1:** Demographic and clinicopathological characteristics of patient cohort

Characteristics (n = 55)		n (%)
**Ethnicity**		
Chinese		50 (90.9)
Others		5 (9.1)
**Smoking status**		
Never-smoker		52 (94.5)
Smoker		3 (5.5)
**Temporal occurrence** of lung and breast cancer diagnosis		
Lung cancer occurred first		5 (9.1)
Breast cancer occurred first		38 (69.1)
Synchronous (within 6 months)		12 (21.8)
**Family cancer history **(any primary)		
First degree		27 (49.1)
Second degree		4 (7.3)
No known history		24 (43.6)
**Family history of breast and/or lung cancer**		
First degree		8 (14.5)
Second degree		2 (3.6)

Breast cancer		Lung cancer	
Diagnosis year	1976–2018	Diagnosis year	2005–2018
Median age, years (range)	55 (34–81)	Median age, years (range)	65 (48–78)
**Histology**	**n (%)**	**Histology**	**n (%)**
Ductal carcinoma in situ (DCIS)	5 (9.1)	Adenocarcinoma (ADC)	44 (80)
Infiltrating ductal carcinoma (IDC)	33 (60)	Neuroendocrine carcinoma	
Infiltrating lobular carcinoma (ILC)	4 (7.3)	Carcinoid	2 (3.6)
DCIS + DCIS (bilateral)	1 (1.8)	SCLC	3 (5.5)
IDC + DCIS (bilateral)	2 (3.6)	Undifferentiated	1 (1.8)
IDC + ILC (bilateral)	2 (3.6)	Lymphoepithelioma-like carcinoma (LELC)	1 (1.8)
IDC + unknown subtype (bilateral)	1 (1.8)	ADC + Carcinoid (bilateral, synchronous)	1 (1.8)
Mucinous adenocarcinoma	3 (5.5)	ADC + ADC (bilateral, synchronous)	1 (1.8)
Subtype not specified (NOS)	4 (7.3)	ADC + ADC (same side, synchronous)	2 (3.6)
**Staging**^**a**^		**Staging**^**b**^	
0	6 (10.9)	0	0 (0.0)
I/II	44 (80.0)	I/II	26 (47.3)
III	4 (7.3)	III	7 (12.7)
IV	1 (1.8)	IV	22 (40.0)
**Hormone and HER2 status**^**c**^		**EGFR status (Adenocarcinoma only, n = 49)**	
**ER**		Not tested	4 (8.2)
Positive	36 (65.4)	Tested	45 (91.8)
Negative	10 (18.2)	**Mutant**	33 (73.3*)
Not tested/unknown	9 (16.4)	Exon19 del	19 (57.6^)
**PR**		L858R	11 (33.3^)
Positive	30 (54.5)	Others	3 (9.1^)
Negative	15 (27.3)	**Wild type**	12 (26.7*)
Not tested/unknown	10 (18.2)		
**HER2**			
Positive	4 (7.3)		
Negative	28 (50.9)		
Not tested/unknown/equivocal	23 (41.8)		

*Over Tested cases (total n = 45)

^^^Over Mutant cases (total n = 33)

^a^For bilateral cancers, higher stage was taken

^b^All 4 multi-lesion cases are stage I

^c^For bilateral cancers, higher stage’s status was presented

### ClinVar-recorded pathogenic or likely pathogenic variants

**Fig. 2:**
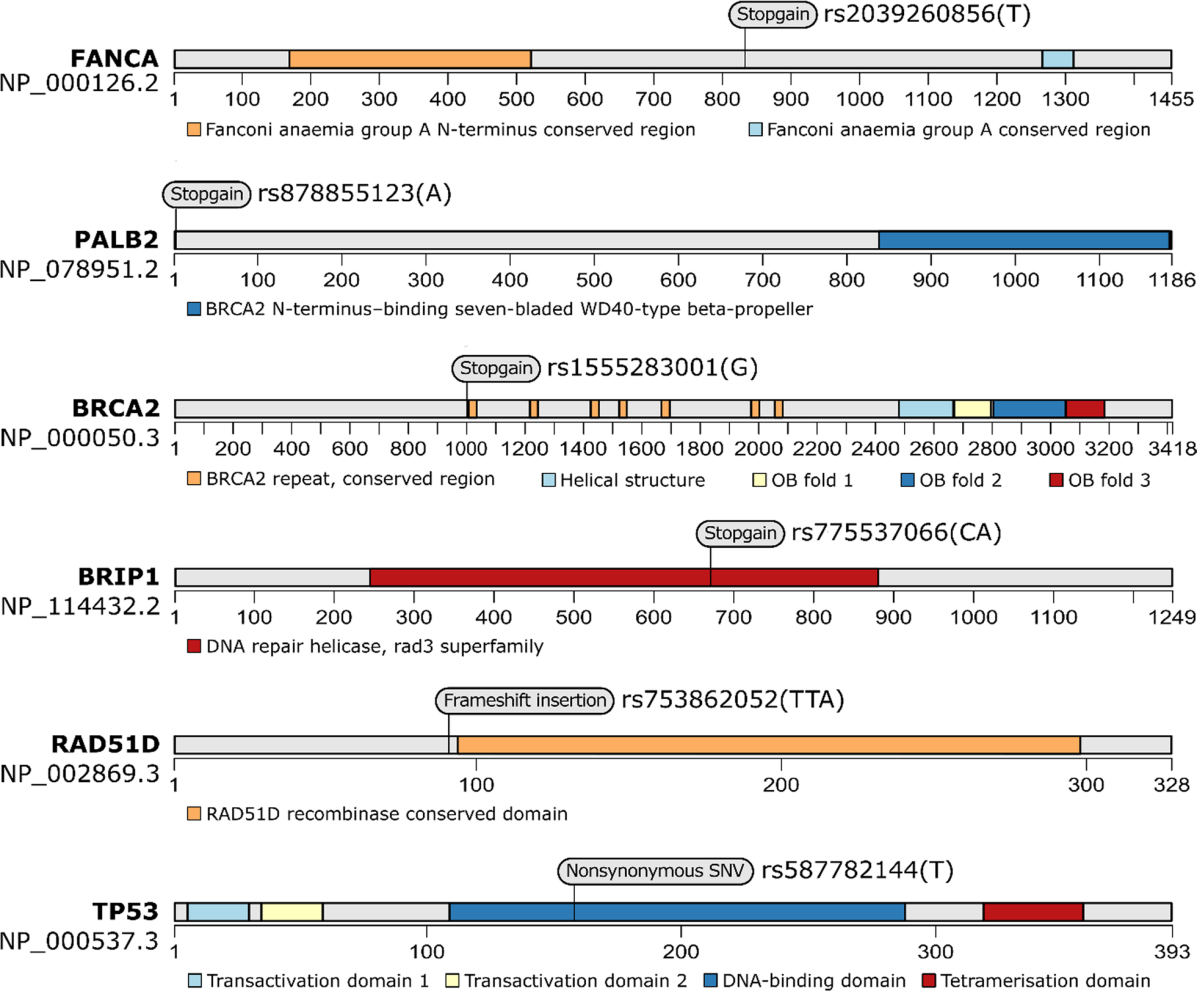
ClinVar-recorded pathogenic or likely pathogenic variants found in 55 patients with dual breast and lung cancer

We identified six variants in five patients which were recorded as pathogenic or likely pathogenic variants by multiple submitters on ClinVar in the context of cancer predisposition syndromes (Fig. 2). Expectedly, these six variants were all in known cancer genes related to DNA damage repair (gene set "DNA repair" in the Gene Ontology database): *FANCA*, *PALB2*, *BRCA2*, *BRIP1*, *RAD51D*, and *TP53*.

### Newly classified pathogenic or likely pathogenic variants

Applying the ACMG/AMP guidelines [11] on all variants except for the six ClinVar-recorded pathogenic or likely variants, we newly classified seven pathogenic variants and six likely pathogenic variants in 13 genes, in 12 patients (Table 2). These guidelines include evidence of pathogenicity from computational and predictive data (Additional file 1: Tables S2–S5), as well as from population data (Additional file 1: Table S6). The 12 patients with newly classified pathogenic or likely pathogenic variants do not overlap with the five patients with ClinVar-recorded pathogenic or likely pathogenic variants.

**Table 2:** List of newly classified pathogenic or likely pathogenic variants and their corresponding ACMG-AMP criteria

Gene	Variant	Classification	Consequence		Computational and Predictive Data								Population data						
				PVS1^a^	PS1^a^	PM5^a^	PM4^a^	PP3^a^ (predictions)					PM2	PS4^a^ (enriched in cases)					
								REVEL^b^	CADD^b^	SIFT^b^	MUTA^b^	PLPH^b^		B&L^c^		BR^d^		LU^e^	
														vs GN^f^	vs SG^g^	vs GN^f^	vs SG^g^	vs GN^f^	vs SG^g^
*EXT2*	chr11:g.44109284G > C	Pathogenic	Splice site	✓					✓		✓		✓	**	**				
*BRIP1*	rs1426528935 (T)	Pathogenic	Fr. del	✓					✓		✓			**	**			*	*
*FANCA*	rs755104393 (G)	Pathogenic	Splice site	✓					✓		✓			*	*				
*WWOX*	rs758588684 (C)	Pathogenic	Startloss	✓					✓	✓	✓	✓		*					
*GATA2*	rs1166272013 (G)	Pathogenic	Splice site	✓									✓	**	**				
*ERCC6/PGBD3*	rs181055741 (A)	Pathogenic	Stopgain	✓											*				
*TP53*	rs1019340046 (G)	Pathogenic	Nonsyn. SNV		✓	✓		✓					✓	**	**				
*GPC3*	rs1158430569 (T)	Likely pathogenic	Nonsyn. SNV			✓								*	**				*
*MSH2*	rs768572053 (G)	Likely pathogenic	Nonsyn. SNV			✓								*	*				
*WRN*	rs569405870 (A)	Likely pathogenic	Nonsyn. SNV			✓								*	*				
*PTEN*	chr10:g.87864072TCTA > T	Likely pathogenic	Non-fr. del				✓						✓		*				
*FANCI*	rs747603151 (T)	Likely pathogenic	Non-fr. del				✓							*	*			*	
*KMT2A*	chr11:g.118436698C > CGCG	Likely pathogenic	Non-fr. ins				✓							*	*				

Fr., frameshift; del., deletion; ins., insertion; ✓, the variant of this row satisfies the criteria of this column; *, the variant of this row is enriched in cases over controls at *p* < 0.05; **, the variant of this row is enriched in cases over controls at *p* < 0.01

^a^PVS1, predicted null variant in a gene where loss of function is a known mechanism of disease; PS1, same amino acid change as an established pathogenic variant; PM5, novel missense change at an amino acid residue where a different pathogenic missense change has been seen before; PM4, protein length changing variant; PP3, multiple lines of computational evidence support a deleterious effect on the gene/gene product; PM2, absent in population databases gnomAD v2.1.1 non-cancer East Asians (EAS) and SG10K_Health; PS4, prevalence in affected statistically increased over controls

^b^REVEL, if all protein changes have REVEL score greater than 0.68; CADD, if the scaled CADD score is greater than 20; SIFT, if SIFT predicts that this variant is pathogenic; MUTA, if MutationTaster predicts that this variant is pathogenic; PLPH, if PolyPhen-2 HDIV or HVAR predicts that this variant is pathogenic

^c^B&L, the cohort of 55 patients with dual breast and lung cancer

^d^BR, the cohort of 290 early-onset or familial breast cancer patients from Singapore

^e^LU, the cohort of 209 lung cancer adenocarcinoma patients from Singapore

^f^vs GN, in a Fisher’s Exact Test of allele frequencies against 9,626 non-cancer exomes and whole-genomes of the gnomAD v2.1.1 East Asian (EAS) subpopulation

^g^vs SG, in a Fisher’s Exact Test of allele frequencies against 9,770 whole-genomes from healthy Singaporean volunteers from SG10K_Health

As with the ClinVar-recorded variants, most of these genes are well-known cancer genes or DNA damage repair genes: *TP53*, *MSH2*, *FANCA*, *ERCC6, BRIP1, WRN*, *FANCI*, *KMT2A,* and *PTEN* (Additional file 1: Table S7); though some are lesser known: *EXT2*, *WWOX*, *GATA2*, and *GPC3.* The *EXT2, WWOX, and GATA2* variants were backed by very strong (“PVS1”) computational and predictive data, as there are pathogenic stop-gain variants for each gene in ClinVar associated with hereditary cancer-predisposing syndrome, malignant tumor of the esophagus, and susceptibility to acute myeloid leukemia, respectively (Additional file 1: Table S2). The *GPC3* variant was backed by moderate (“PM5”) computational and predictive data as it modifies the same amino acid residue as another pathogenic missense variant in ClinVar associated with Wilms tumor (Additional file 1: Table S4).

Despite each variant being present in only one heterozygous patient in the dual primary breast and lung cohort of 55 patients, they were all enriched in that case cohort when compared to gnomAD non-cancer East Asians (n = 9626) or SG10K_Health, a local cohort of healthy volunteers (n = 9770) as per the ACMG/AMP guidelines. As expected of rare variants, only five of these 13 variants were also detected in a local cohort of lung cancer patients (n = 209) of which only three were enriched in that lung cancer cohort versus the two control cohorts. However, none of these 13 variants could be found in a local cohort of early-onset or familial breast cancer patients (n = 290).

Some variants satisfied at least one pathogenicity criteria, but not enough to be classified as pathogenic or likely pathogenic. In total, there are 33 such variants of uncertain significance (VUS) (Additional file 1: Table S8).

### Clinical features of patients with pathogenic or likely pathogenic variants

Altogether, 17 of 55 (31%) patients had pathogenic or likely pathogenic variants (Table 3). Of these, 6 variants, which are clinically annotated as pathogenic/likely pathogenic in ClinVar, were detected in 9% (5/55) of patients, and 13 newly classified variants were found in 22% (12/55) of patients (Additional file 1: Table S9). Notably, each of these variants is unique to a single patient, and 2 patients were carriers for two variants each: Patient H3168 had stop gain variants in *FANCA* and *PALB2*, and had a sister with lung cancer, and another sister with breast cancer. Patient H4326 had a nonsynonymous variant in *MSH2* and a non-frameshift deletion in *PTEN*, and had a family history of two sisters having lung cancer. There was no observed association between the presence of pathogenic or likely pathogenic variants and EGFR mutation status (Additional file 1: Table S10).

**Table 3:** Clinical features of 17 patients with dual primary breast and lung cancer with pathogenic or likely pathogenic variants

Patient ID^a^	Gene	Variant	Consequence	Family History (Age at Dx)	Breast cancer			Lung cancer				Temporal occurrence of Br and Lung Ca Dx
					Age at Dx (years)	Histo type (Laterality)	ER/PR/Her2 status	Age at Dx (years)	Histo type	Smoking history	EGFR status	
H3168	*FANCA*	rs2039260856 (T)	Stop gain	Sis Lung Ca (> 50); Sis Br Ca (58); Fa HNSCC (62)	45	ILC (L)	Unk	66	ADC	None	E746_A75del	Br before Lung Ca
	*PALB2*	rs878855123 (A)	Stop gain									
H1641	*BRCA2*	rs1555283001 (G)	Stop gain	Mo Pancreatic Ca (70 +)	56	IDC (L)	ER + /PR-/Her2 +	64	ADC	None	E746_A75del	Br before Lung Ca
H1362	*BRIP1*	rs775537066 (CA)	Stop gain	No known	60	IDC (L)	ER-/PR-/Her2 +	63	LELC	None	Unk	Br before Lung Ca
H1409	*RAD51D*	rs753862052 (TTA)	Frameshift ins	Mo Col Ca (Unk); Sis Ov Ca (Unk)	68	IDC (R)	ER + /PR-/Her2-	68	ADC	None	WT	Synchronous
H2590	*TP53*	rs587782144 (T)	Nonsyn. SNV	No known	49	NOS (R), IDC (L)	Unk (R), ER + /PR-/Her2- (L)	74	ADC	None	E746_A75del	Br before Lung Ca
H3587	*EXT2*	chr11:g.44109284G > C	Splice site	Bro Pros Ca (Unk)	53	IDC (R)	ER + /PR + /Her2-	60	LCNEC	None	Unk	Br before Lung Ca
H1026	*BRIP1*	rs1426528935 (T)	Frameshift del	Fa Lung Ca (Unk); Mo Lung Ca (Unk); Sis Br Ca (Unk)	46	NOS (L)	Unk	55	ADC	None	E746_A75del	Br before Lung Ca
H3539	*FANCA*	rs755104393 (G)	Splice site	Niece Lung Ca (40 +)	63	IDC (R)	ER + /PR + /Her2-	75	ADC	None	N771_P772insPH	Br before Lung Ca
H4324	*WWOX*	rs758588684 (C)	Start loss	No known	47	IDC (R)	Unk	63	ADC	None	WT	Br before Lung Ca
H2552	*GATA2*	rs1166272013 (G)	Splice site	No known	59	DCIS (R)	ER + /PR-/Her Unk	59	ADC	None	WT	Synchronous
H3400	*ERCC6/PGBD3*	rs181055741 (A)	Stop gain	Bro Pros Ca (63); Fa HNSCC (70)	62	MADC (L)	ER + /PR + /Her2 +	62	ADC	None	E746_A75del	Synchronous
H4270	*TP53*	rs1019340046 (G)	Nonsyn. SNV	Fa Gastric Ca (40)	34	DCIS (R), IDC (L)	Unk (R), ER + /PR + /Her2 Unk (L)	52	SCLC	None	WT	Br before Lung Ca
H1758	*GPC3*	rs1158430569 (T)	Nonsyn. SNV	No known	68	IDC (R)	ER + /PR + /Her2 Equiv	69	ADC	None	L858R	Br before Lung Ca
H4326	*MSH2*	rs768572053 (G)	Nonsyn. SNV	Sis Lung Ca (60); Sis Lung Ca (69)	64	DCIS (R)	ER + /PR + /Her2 Unk	59	ADC	Former	WT	Lung before Br Ca
	*PTEN*	chr10:g.87864072TCTA > T	Non-frameshift del									
H3053	*WRN*	rs569405870 (A)	Nonsyn. SNV	No known	65	IDC (R)	ER + /PR + /Her2-	65	ADC	None	WT	Synchronous
H2235	*FANCI*	rs747603151 (T)	Non-frameshift del	Sis Ute Ca (40 +); Fa Unk (60–70)	67	IDC (R), ILC (R)	ER + /PR + /Her2-	74	ADC	None	WT	Br before Lung Ca
H2420	*KMT2A*	chr11:g.118436698C > CGCG	Non-frameshift ins	No known	63	IDC (L)	ER + /PR + /Her2-	72	ADC	None	Exon19del	Br before Lung Ca

ADC, adenocarcinoma; Br, breast; Bro, brother; C, Chinese; Ca, cancer; Col, colorectal; DCIS, ductal carcinoma in-situ; Del., deletion; Dx, diagnosis; EGFR, epidermal growth factor receptor; Equiv, equivocal; ER, oestrogen receptor; Fa, father; Histo, histological; HNSCC, head and neck squamous cell carcinoma; IDC, invasive ductal carcinoma; ILC, invasive lobular carcinoma; Ins., insertion;L, left; LCNEC, large cell neuroendocrine lung carcinoma; LELC, lymphoepithelioma-like carcinoma; Li, liver; MADC, mucinous adenocarcinoma; Mo, mother; Nonsyn., non-synonymous; NOS, not otherwise specified; NPC, nasopharyngeal cancer; NSCLC, non-small cell lung cancer; Ov, ovarian; PR, progesterone receptor; Pros, prostate; R, right; SCLC, small cell lung cancer; Sis, sister; SNV, single nucleotide variant; Unk, unknown; Ute, uterine; WT, wildtype

^a^Patients in the breast and lung cohort with variants, all of whom are heterozygous for their corresponding variants

Stop gain and nonsynonymous variants were the most prevalent among our patient cohort, followed by splice site variants. Among patients with pathogenic/likely pathogenic variants, 59% (10/17) of patients had a family history of cancer. Specifically, only 4 patients had a family history of breast and/or lung cancer. Sixteen of our 17 patients identified as ethnic Chinese, with one patient, H2552, who is of Indian ethnicity. Detailed clinical features of the 17 patients with pathogenic/likely pathogenic variants are presented in Table 3.

## Discussion

There are limited WGS or WES studies on patients with multiple primary cancers. To the best of our knowledge, we report here the largest WES on patients with dual primary cancers of the breast and lung. Overall, our study uncovered that 31% (17/55) of our patients with dual primary breast and lung cancer harbor pathogenic or likely pathogenic variants in cancer associated genes.

The majority of the patients in our cohort had developed breast cancer before lung cancer. This is in concordance with the findings of other studies that have observed that primary lung cancer occurred after a previous primary breast cancer [10], which may be related to the higher survival probability after diagnosis of breast cancer compared to lung cancer. The interval between the development of a second primary lung cancer after breast cancer was less than 10 years in 25.5% of our cohort, while 34.5% of our patients developed lung cancer 10 to 20 years later. This was in contrast to a WES study comprising 60 breast cancer survivors from Italy, where a second primary lung cancer developed after approximately 7 years [7]. That cohort had 46.7% smokers whereas 94.5% of our cohort were never-smokers.

Radiotherapy may result in increased risk of subsequent lung cancer on the same side as the breast cancer where the radiation was administered [3, 4], though the risk may be lower with modern radiotherapy techniques. Of 194,981 women with nonmetastatic invasive breast cancer who had been treated with mastectomy, women who had also been treated with radiotherapy had a moderate increased risk for developing ipsilateral lung carcinoma after 10 years, but there was no increased risk for the contralateral lung [12]. Of the 11 patients in our study who had received radiotherapy for the treatment of breast cancer, only 6 had ipsilateral breast and lung cancer. Thus, the lack of predominance of patients having received radiotherapy later developing lung cancer on the same side supports the fact that these cancers arose independently without this "iatrogenic cause". Hence other factors such as intrinsic genetic predisposition may play a role in the development of these lung cancers.

There is an ongoing unmet need in identifying risk alleles for lung and breast cancers to optimize genetic screening strategies. This is especially important in lung cancer screening in Asia where ongoing efforts to determine the optimal strategy especially in “at risk” never smokers. A recent study has identified germline pathogenic variants in 4.3% (222/5,118) of patients who had undergone genomic profiling. Of these 222 patients, pathogenic variants in DNA damage repair pathway genes were detected in 193 patients, including in *BRCA2* (n = 54) [13]. Germline susceptibility genes warrant further investigation as to their role in risk prediction models.

Among the 55 patients in our cohort who had dual cancers in the breast and lung, we identified variants in 10 DNA damage repair genes in 12 patients, which included *FANCA, PALB2, BRCA2*, *BRIP1, RAD51D, TP53, ERCC6, MSH2, WRN*, and *FANCI*, with *PALB2, BRCA2, RAD51D*, *TP53* and *PTEN* being known breast cancer susceptibility genes [14–17]. Germline variants in many of these genes are frequently observed in cancer patients referred for genetic testing, including those with multiple primary malignancies. For example, Lynch syndrome is often associated with pathogenic variants in *MSH2*, while pathogenic variants in *BRCA1* and *BRCA2* are commonly associated with hereditary breast and ovarian cancer [15, 18, 19].

Our study extends the findings of previous research proposing that DNA damage repair pathways contribute to cancer development, including in breast and lung cancers [20]. Specifically, our results suggest that DNA damage repair pathways may play a critical role in the development of dual primary breast and lung cancer, as a significant proportion of the variants we found are implicated in DNA repair. For example, the *FANCA* and *FANCI* genes which have been implicated in breast cancer, had pathogenic variants detected in both breast cancer patients and individuals with hereditary breast and ovarian cancer (HBOC) [21–24]. We also identified pathogenic variants in other DNA repair genes such as *WRN* and *ERCC6*. *WRN* is linked to Werner syndrome, a rare disease characterized by premature aging and increased predisposition to cancer. Multiple studies have reported an association between *WRN* variants and hereditary breast cancer as well as HBOC [23, 25, 26]. Additionally, germline variants and genetic polymorphisms in the *ERCC6* gene have been associated with early onset breast cancer and an elevated risk of lung cancer, respectively [27, 28].

The exact mechanism by how germline variants in DNA damage repair genes lead to MPCs remains unclear. However, exposure to carcinogens and treatments like chemotherapy and radiation used to treat the first cancer can exacerbate the effects of these variants, increasing the risk of developing a second primary cancer [29]. A recent study has also established associations between air pollution and lung cancer in non-smokers or light smokers [30].

In addition, our study identified variants in several lesser-known cancer genes, including *EXT2*, *WWOX*, *GATA2*, and *GPC3*. To the best of our knowledge, the specific germline variants found in our study have not been previously reported in any cancer-related studies. However, other germline alterations have been detected in these genes. For example, in *EXT2*, germline variants (NP_000392.3:p.Trp79* and NP_997005.1:p.Trp46*) are associated with hereditary multiple osteochondromas, a non-cancerous condition that can develop into chondrosarcoma [31]. Another study reported that germline variants (p.W606X, IVS1762-1G > A, p.T507fs, and p.T642fs) in *EXT2* were found in 4 out of 1026 patients with non-small cell lung cancer [32]. Interestingly, a female patient with early-onset multiple primary tumors was reported to have a germline homozygous *WWOX* variant (NC_000016.9:g.79245877_79245881dup) [33]. Multiple germline variants in *GATA2* predispose individuals to familial myelodysplastic/acute leukemia syndrome [34, 35], while germline *GPC3* variants are observed in Simpson-Golabi-Behmel syndrome, an overgrowth syndrome that increases the risk of developing Wilms tumor, hepatoblastoma, and neuroblastoma [36, 37].

None of the 19 pathogenic or likely pathogenic variants identified in the dual primary breast and lung cohort were found in the local cohort of 290 early-onset or familial breast cancer patients (Additional file 1: Table S6). This latter cohort comprised of *BRCA*-negative cases. The absence of these dual primary breast and lung variants in the breast cancer cohort might be explained by the high genetic heterogeneity of these variants, with each of these 19 variants detected in only one dual breast and lung cancer patient. Such high genetic heterogeneity has been observed in *BRCA*-negative familial high-grade serous ovarian carcinoma patients, where WES had detected rare loss-of-function variants that occurred in less than 0.5% of individuals [38]. Our findings suggest that patients with dual breast and lung cancer could have additional genes involved in cancer predisposition, other than genes known to be associated with breast cancer predisposition.

We have compared our variants against those detected by other studies on MPCs. One study had performed WES on 28 of 60 patients from Italy with breast and lung cancer, and had identified one gene, *GSN,* to be significantly enriched [7]. However, *GSN* variants were not observed in our study. This discrepancy could be attributed to differences in the ethnic distribution between our study and the previous study, or to the small sample sizes of both studies highlighting the impact of genetic heterogeneity on variant detection. A second study on patients with MPCs had focused only on 83 known cancer predisposition genes. However, pathogenic variants in these 83 genes were not observed in our patients with primary breast and lung cancer [8]. In a third study, gene-based burden testing showed that there was an increase in carriers with *BRCA1* and *BRCA2* loss of function variants for breast cancer patients with an additional lung cancer [9]. A pathogenic *BRCA2* variant was also found in one of our 55 patients.

Our study has also identified a high rate of VUS, with 63% (33/52) of variants detected in 31 out of 55 patients (56%) patients (Additional file 1: Table S9). This is comparable to the overall VUS rate of 49.9% reported in another cohort of Singaporean patients who underwent genetic testing [39]. These findings align with our previous studies on Asian populations, which have consistently shown higher VUS rates compared to populations of European descent, possibly due to the underrepresentation of Asians in reference databases [40–42]. However, as more data becomes available, VUS can be reclassified [43, 44]. Previous studies have reported VUS reclassification rates of 6–15%, with a Singaporean study reporting a reclassification rate of 6.7% over a 6-year period [39]. Of these reclassifications, 94.1% of VUS were downgraded to benign or likely benign variants, while 5.9% were upgraded to pathogenic or likely pathogenic variants. Such reclassifications have a significant impact on the risk assessment and management of patients with suspected or confirmed hereditary cancer, particularly in understudied populations.

A limitation of this study is the small sample size (n = 55) utilized in this cohort, which limits the statistical power of the study, and this may have led to no associations observed between the occurrence of our pathogenic variants and clinicopathological features. However, this is inevitable since the occurrence of dual primary breast and lung cancer is relatively uncommon. Another limitation was that we were unable to perform segregation analysis and validation analysis on additional patients with primary cancers of both the breast and lung, due to the unavailability of DNA samples. Therefore, confirmation of the variants identified in this study in larger cohorts of patients with dual cancers of the breast and lung is warranted.

## Conclusions

In summary, through employing WES, we have identified variants that are significantly enriched in patients with primary malignancies in both the breast and lung. This study represents the largest cohort of patients with multiple primaries in the breast and lung, that have been sequenced. Altogether, we have identified 19 pathogenic or likely pathogenic variants in 16 genes, detected in 17 patients. Ten of these 16 genes are DNA damage repair genes, underscoring the importance of these genes in patients with dual malignancies of the breast and lung.

## Materials and methods

### Patient samples

An overview of our study design is shown in Fig. 1. Peripheral blood or saliva samples were obtained from 55 patients with primary breast and primary lung cancer from the National Cancer Centre Singapore. The later-occurring cancer was verified as a primary cancer by histopathology. Written informed consent was obtained from all patients and the study was approved by the SingHealth Centralised Institutional Review Board (CIRB Ref: 2018/2147 and 2018/2963). The samples were retrospectively selected.

### Whole-exome sequencing (WES), quality control, and variant annotation

Genomic DNA was extracted from blood or saliva samples. Saliva samples were used when blood samples were not available. Altogether, 15 of our 55 cases used DNA extracted from saliva samples. The Agilent SureSelect Human All Exon V6 kit (Agilent Technologies, CA, USA) was used to prepare sequencing libraries from DNA samples and the Illumina Novaseq 6000 platform was used for 150 bp paired-end sequencing of up to 100X by a service provider, Novogene AIT Singapore.

For germline variant calling, Genome Analysis Toolkit (GATK) v4.2.6.0 with the hg38 reference genome was used [45]. Each sample was individually aligned, recalibrated for improved quality scores using GATK’s Base Quality Score Recalibration (BQSR), individually re-assembled to form haplotypes (GVCF files), then jointly-called to obtain genotypes (VCF file). Thereafter, technical artifacts were removed using GATK’s Variant Quality Score Recalibration (VQSR). In addition, all case variants reported in this study were visually checked with Integrative Genomics Viewer (IGV), and variants with low read depths or imbalanced variant allele frequencies were omitted. Case variants were annotated for functional consequence using ANNOVAR [46], REVEL v1.3 using pre-computed scores, [47] CADD v1.6 scores using the CADD offline scoring program [48], SIFT 4G using the pre-computed SIFT 4G annotator [49], PolyPhen-2 using the pre-computed variant call format (VCF) database file [50], and MutationTaster using the MutationTaster2021 webserver [51]. Case variants were lifted-over from hg38 to hg19 using GATK LiftoverVcf (Picard) with UCSC chain files for PolyPhen-2 and MutationTaster annotation as these services only supported hg19.

### Identification of ClinVar pathogenic or likely pathogenic variants

The weekly release of the ClinVar database dated 18 February 2023 was used. Relevant cancer conditions or cancer predisposition syndromes were manually selected from the list of all conditions in ClinVar (Additional file 1: Table S11). Variants were defined as pathogenic and likely pathogenic variants when the variants were interpreted as pathogenic or likely pathogenic by an expert panel, practice guidelines, or multiple submitters with named criteria and without conflicting interpretations. Hereafter, only pathogenic and likely pathogenic variants satisfying these levels of evidence in this list of relevant conditions are considered in our analysis as “ClinVar variants”; while other variants such as those evidenced only by single submitters or with conflicting interpretations are ignored.

### ACMG/AMP computational and predictive data criteria

Case variants were evaluated for five criteria within the ACMP/AMP group of Computational and Predictive data criteria as described previously [11].

For PVS1, defined as a predicted null variant in a gene where loss of function is a known mechanism of disease, we first selected genes with pathogenic ClinVar variants functionally annotated by ANNOVAR as splice site changes, frameshift indels, start-losses, or stop-gains. Then, we selected case variants with the same functional annotations, also in those selected genes.

For PS1, defined as the same amino acid change as an established pathogenic variant, we selected case variants sharing at least one amino acid change with a pathogenic ClinVar variant: the case variant and ClinVar variants must change the same residue to the same amino acid.

For PM5, defined as a novel missense change at an amino acid residue where a different pathogenic missense change has been seen before, we selected case variants sharing at least one modified residue with a pathogenic ClinVar variant: the case variant and ClinVar variant must change the same residue, but to different amino acids.

For PM4, defined as a protein length changing variant, we selected case variants functionally annotated as non-frameshift indels in non-repeat regions (identified by the UCSC Genome Browser track “simpleRepeats,” last updated 18 October 2022) or as stop-losses, in genes with at least one pathogenic or likely pathogenic ClinVar variant.

For PP3, defined as a variant having multiple lines of computational evidence supporting a deleterious effect on the gene/gene product, we selected case variants with no benign predictions and at least two pathogenic/deleterious predictions from REVEL, CADD, SIFT, PolyPhen-2 (HumDiv-trained Polyphen-2 model or HumVar-trained Polyphen-2 model), and MutationTaster. For REVEL, a genomic variant was considered pathogenic if all its protein changes had REVEL scores greater than 0.68, corresponding to a specificity of 95%); or benign if any of its protein changes had REVEL scores less than or equal to 0.32, for symmetry with the 0.68 pathogenicity threshold. For CADD, a genomic variant was considered pathogenic if it had PHRED-scaled score greater than 20, corresponding to the top 0.1% of variants by pathogenicity.

### ACMG/AMP population data criteria

Case variants satisfying any of the five pathogenicity criteria above were further evaluated for three criteria within the ACMP/AMP group of Population Data criteria.

For BS1, defined as a variant having a minor allele frequency (MAF) that is too high for a disorder, we excluded variants with a minor allele frequency greater than 1% in either gnomAD (EAS) or SG10K_Health control cohorts. The gnomAD (EAS) control cohort refers to the 9,626 non-cancer exomes and whole-genomes of the gnomAD v2.1.1 East Asian (EAS) subpopulation [52]. The SG10K_Health control cohort comprises the whole genome sequences of 9,770 healthy Chinese, Indian, and Malay Singaporeans [53].

For PM2, defined as a variant that is absent in population databases, we selected variants not recorded in both gnomAD (EAS) and SG10K_Health control cohorts. This is different and more conservative than selecting variants with zero allele count, as a variant can be recorded with zero allele count in the EAS subpopulation if it is present in any other gnomAD subpopulation. Instead, an unrecorded variant in gnomAD (EAS) represents a variant not found in any gnomAD subpopulation.

For PS4, defined as a variant having a prevalence in affected individuals that is statistically increased over controls, we select variants statistically enriched in cases at *p* < 0.05, in at least one of six pairwise case–control association analyses between three case cohorts and the two control cohorts gnomAD (EAS) and SG10K_Health; but exclude any variant statistically enriched in controls in any of the six comparisons.

The three case cohorts used are: (1) the 55 patients with dual primary breast and lung cancers, subjected to WES in this study; (2) a cohort of 290 patients with early-onset (≤ 40 years old) or familial breast cancer from Singapore [40]; and (3) a lung cancer case cohort of 209 lung adenocarcinoma patients from Singapore [54].

Case–control association analyses were performed using a two-tailed Fisher’s Exact Test. To enable the statistical test of unrecorded variants in the two control cohorts, allele numbers (the number of alleles that were successfully genotyped at that position) were linearly interpolated from the two closest variants within 150 bp upstream and downstream of the unrecorded variant.

### Identification of newly classified pathogenic or likely pathogenic variants

Following the ACMG/AMP guideines [11] case variants were classified as pathogenic if they satisfied pathogenicity criteria PVS1 and PS4, or PS1 and PS4. Case variants were classified as likely pathogenic if they satistified pathogenicity criteria PVS1 and PM2, PS1 and PM2, PS4 and PM4, or PS4 and PM5. Variants which satisfied some pathogenicty criteria but could not be classified as pathogenic or likely pathogenic were classified as variants of uncertain significance. Any variant satisfying benign criterion BS1 was excluded from consideration.

### Statistical analysis, software, and additional databases

The Fisher’s Exact Test was performed using the *R* stats package [55]. The protein domains shown for illustration were obtained from the NCBI Conserved Domain Database, accessed 11 April 2023 [56]. Pathways were obtained using the online “Investigate Human Gene Sets” tool of the Gene Set Enrichment Analysis website, computing overlaps with the Gene Ontology Biological Process database without correction for the universe set of genes and pathways, accessed 12 April 2023 [57].

### Supplementary Information

**Additional file 1. Table S1**: Comparison of breast cancer statuses in EGFR-mutated versus non-EGFR-mutated lung adenocarcinoma patients. **Table S2**. List of well-evidenced ClinVar null variants in genes with PVS1 variants. **Table S3**. List of ClinVar variants with the same amino acid change as PS1 variants. **Table S4**. List of ClinVar variants with the same modified residue as PM5 variants. **Table S5**. Pathogenicity predictions for various in silico prediction software for PP3 variants. **Table S6**. Frequency and Fisher's Exact Test results for three case cohorts and two control cohorts, for all pathogenic or likely pathogenic variants from this study. **Table S7**. Pathways of genes from all pathogenic and likely pathogenic variants in this study. **Table S8**. List of variants of uncertain significance and their corresponding ACMG/AMP criteria. **Table S9**. The number of variants of each type detected in each patient with dual primary breast and lung cancer. **Table S10**. Lack of association between the presence of pathogenic or likely pathogenic variants, and EGFR mutation statuses. **Table S11**: Cancers and cancer predisposition syndromes in ClinVar and their associated genes.

## Data Availability

The gnomAD dataset was analyzed in this study and is accessible at https://gnomad.broadinstitute.org/. Access to the SG10K_Health dataset can be requested from the Singapore National Precision Medicine program. The analysis pipeline used to generate these results is accessible at https://github.com/ning-y/nccs-lcc4.

## Abbreviations

ACMG/AMP: American College of Medical Genetics and Association for Molecular Pathology
EAS: East-Asian
GATK: Genome analysis toolkit
gnomAD: Genome aggregation database
IGV: Integrative genomics viewer
MAF: Minor allele frequency
MPCs: Multiple primary cancers
VCF: Variant call format
VUS: Variants of uncertain significance
WES: Whole-exome sequencing
WGS: Whole-genome sequencing
