# Correction: Landscape of germline pathogenic variants in patients with dual primary breast and lung cancer

**DOI:** 10.1186/s40246-023-00518-z

**Published:** 2023-08-14

**Authors:** Ning-Yuan Lee, Melissa Hum, Sabna Zihara, Lanying Wang, Matthew K. Myint, Darren Wan-Teck Lim, Chee-Keong Toh, Anders Skanderup, Jens Samol, Min-Han Tan, Peter Ang, Soo-Chin Lee, Eng-Huat Tan, Gillianne G. Y. Lai, Daniel S. W. Tan, Yoon-Sim Yap, Ann S. G. Lee

**Affiliations:** 1 Division of Cellular and Molecular Research, National Cancer Centre Singapore, 30 Hospital Boulevard, Singapore, 168583 Singapore; 2 Division of Medical Oncology, National Cancer Centre Singapore, 30 Hospital Boulevard, Singapore, 168583 Singapore; 3 SingHealth Duke-NUS Oncology Academic Clinical Programme (ONCO ACP), Duke-NUS Medical School, 8 College Road, Singapore, 169857 Singapore; 4 Genome Institute of Singapore, 60 Biopolis St, Singapore, 138672 Singapore; 5 Medical Oncology Department, Tan Tock Seng Hospital, 11 Jalan Tan Tock Seng, Singapore, 308433 Singapore; 6 Johns Hopkins University, Baltimore, MD 21218 USA; 7 Lucence Diagnostics Pte Ltd, 211 Henderson Road, Singapore, 159552 Singapore; 8 Oncocare Cancer Centre, Gleneagles Medical Centre, 6 Napier Road, Singapore, 258499 Singapore; 9 Department of Hematology-Oncology, National University Cancer Institute, Singapore (NCIS), National University Health System, 5 Lower Kent Ridge Road, Singapore, 119074 Singapore; 10 Cancer Science Institute, Singapore (CSI), National University of Singapore, 14 Medical Dr, Singapore, 117599 Singapore; 11 Clinical Trials and Epidemiological Sciences, National Cancer Centre Singapore, 30 Hospital Boulevard, Singapore, 168583 Singapore; 12 Department of Physiology, Yong Loo Lin School of Medicine, Medical Drive, National University of Singapore, Singapore, 117593 Singapore

**Correction: Human Genomics (2023) 17:66** 10.1186/s40246-023-00510-7

Following publication of the original article [1], the authors reported that some words in Table 1 were out of alignment and it needed to be corrected.

The correct Table 1 has been provided in this correction.

**Table 1:** Demographic and clinicopathological characteristics of patient cohort

Characteristics (n = 55)		n (%)
**Ethnicity**		
Chinese		50 (90.9)
Others		5 (9.1)
**Smoking status**		
Never-smoker		52 (94.5)
Smoker		3 (5.5)
**Temporal occurrence** of lung and breast cancer diagnosis		
Lung cancer occurred first		5 (9.1)
Breast cancer occurred first		38 (69.1)
Synchronous (within 6 months)		12 (21.8)
**Family cancer history** (any primary)		
First degree		27 (49.1)
Second degree		4 (7.3)
No known history		24 (43.6)
**Family history of breast and/or lung cancer**		
First degree		8 (14.5)
Second degree		2 (3.6)

Breast cancer		Lung cancer	
Diagnosis year	1976–2018	Diagnosis year	2005–2018
Median age, years (range)	55 (34–81)	Median age, years (range)	65 (48–78)
**Histology**	**n (%)**	**Histology**	**n (%)**
Ductal carcinoma in situ (DCIS)	5 (9.1)	Adenocarcinoma (ADC)	44 (80)
Infiltrating ductal carcinoma (IDC)	33 (60)	Neuroendocrine carcinoma	
Infiltrating lobular carcinoma (ILC)	4 (7.3)	Carcinoid	2 (3.6)
DCIS + DCIS (bilateral)	1 (1.8)	SCLC	3 (5.5)
IDC + DCIS (bilateral)	2 (3.6)	Undifferentiated	1 (1.8)
IDC + ILC (bilateral)	2 (3.6)	Lymphoepithelioma-like carcinoma (LELC)	1 (1.8)
IDC + unknown subtype (bilateral)	1 (1.8)	ADC + carcinoid (bilateral, synchronous)	1 (1.8)
Mucinous adenocarcinoma	3 (5.5)	ADC + ADC (bilateral, synchronous)	1 (1.8)
Subtype not specified (NOS)	4 (7.3)	ADC + ADC (same side, synchronous)	2 (3.6)
**Staging**^**a**^		**Staging**^**b**^	
0	6 (10.9)	0	0 (0.0)
I/II	44 (80.0)	I/II	26 (47.3)
III	4 (7.3)	III	7 (12.7)
IV	1 (1.8)	IV	22 (40.0)
**Hormone and HER2 status**^**c**^		**EGFR status** (adenocarcinoma only, n = 49)	
**ER**		Not tested	4 (8.2)
Positive	36 (65.4)	Tested	45 (91.8)
Negative	10 (18.2)	**Mutant**	33 (73.3*)
Not tested/unknown	9 (16.4)	Exon19 del	19 (57.6^)
**PR**		L858R	11 (33.3^)
Positive	30 (54.5)	Others	3 (9.1^)
Negative	15 (27.3)	**Wild type**	12 (26.7*)
Not tested/unknown	10 (18.2)		
**HER2**			
Positive	4 (7.3)		
Negative	28 (50.9)		
Not tested/unknown/equivocal	23 (41.8)		

^a^For bilateral cancers, higher stage was taken

^b^All 4 multi-lesion cases are stage I

^c^For bilateral cancers, higher stage’s status was presented

*Over tested cases (total n = 45)

^^^Over mutant cases (total n = 33)

The original article [1] has been corrected.
